# Retraction: MicroRNA-140-5p suppresses retinoblastoma cell growth via inhibiting c-Met/AKT/mTOR pathway

**DOI:** 10.1042/BSR-2018-0776_RET

**Published:** 2021-06-08

**Keywords:** MiR-140-5p, retinoblastoma, tumorigenesis

This Retraction follows an Expression of Concern relating to this article previously published by Portland Press.

This article is being retracted from *Bioscience Reports* at the request of the Editor-in-Chief and the Editorial Board following receipt of a notification from a reader, alerting the Editorial Board to issues in Figure 3E, which contains areas of duplication in the Retinoblastoma images.

The authors have been contacted in regards to the retraction, and have not responded to the Journal's queries and the concerns raised. Given the extent of the issues raised, the Editorial Board stand by the decision to retract the article.

